# Assessment of behaviors, attitudes, and awareness regarding skin cancer among polish medical and non-medical students: comparative analysis

**DOI:** 10.3389/fonc.2026.1697895

**Published:** 2026-05-08

**Authors:** Natalia Sauer, Katarzyna Romanowicz, Monika Birska, Jakub Staś, Wioletta Dwernicka, Piotr Giedziun, Jacek Calik

**Affiliations:** 1 Department of Clinical Pharmacology, Faculty of Pharmacy, Wroclaw Medical University, Wroclaw, Poland; 2 Faculty of Medicine, Opole University, Opole, Poland; 3 Department of Basic Medical Sciences, Faculty of Pharmacy, Wroclaw Medical University, Wrocław, Poland; 4 Faculty of Medicine, Wroclaw Medical University, Wrocław, Poland; 5 Faculty of Medicine, Poznan University of Medical Sciences, Poznan, Poland; 6 Department of Artificial Intelligence, Wroclaw University of Science and Technology, Wrocław, Poland; 7 Division of Histology and Embryology, Department of Human Morphology and Embryology, Faculty of Medicine, Wrocław Medical University, Wrocław, Poland

**Keywords:** knowledge gaps, medical education, preventive behaviors, public health, skin cancer, sun protection

## Abstract

**Objective:**

This study aimed to evaluate and compare the behaviors, attitudes, and awareness regarding skin cancer among Polish medical and non-medical students to identify gaps and guide the development of targeted educational interventions.

**Methods:**

A cross-sectional survey was conducted among 404 students (58.7% medical, 41.3% non-medical) across Poland between December 2023 and October 2024. Participants completed an online questionnaire assessing demographics, knowledge, attitudes, and sun-protective behaviors. Statistical analyses included chi-squared tests (with Fisher’s exact test where appropriate), Mann–Whitney U tests, and multivariable linear regression to identify predictors of sunscreen use and compare practices across groups.

**Results:**

Medical students exhibited higher knowledge scores (*M* = 6.23, *SD* = 2.07) than non-medical students (*M* = 4.23, *SD* = 2.33), *U = 29321.5*, *p* <.001. However, this difference has been conclusively demonstrated to result from the higher proportion of women among medical students, as statistical analysis confirmed that gender is a significant predictor of sunscreen adherence and knowledge scores. Further analysis using an ordinary least squares (OLS) regression model, *R²* = .120, *F*(4, 399) = 13.58, *p* <.001, showed that when controlling for gender, the academic field (medical vs. non-medical) no longer had a statistically significant effect on sunscreen adherence or knowledge scores (*β* = -0.28, *p* = .109), indicating that the observed differences are fully explained by gender composition rather than academic training. Furthermore, women were significantly more likely to use sunscreen on sunny days (84.7% vs. 61.0%; *χ²*(1, *N* = 404) = 33.18, *p* <.001, φ = .29) and reported higher daily sunscreen use (44.9% vs. 13.0%; *χ²*(1, *N* = 404) = 42.87, *p* <.001, φ = .32). Knowledge scores correlated positively with sunscreen use (*r_s_* = .24, *p* <.001).

**Conclusion:**

Significant disparities in sun protection behaviors exist between genders, with women showing greater adherence to protective measures. The higher knowledge scores among medical students are fully explained by the greater representation of women in this group, as academic field alone does not significantly impact sunscreen adherence or knowledge when controlling for gender. These findings underscore the need for inclusive and targeted educational campaigns to bridge knowledge-practice gaps and improve skin cancer prevention among at-risk populations. Gender-specific interventions, cost reduction strategies, and public health messaging linking sunscreen use to anti-aging benefits may improve adherence, particularly among high-risk populations.

## 1 Introduction

Globally, skin cancer remains one of the most prevalent malignancies, with ultraviolet (UV) radiation exposure serving as the most significant modifiable risk factor (1–3). Despite increased awareness efforts, the incidence of both melanoma and non-melanoma skin cancers continues to rise, reflecting gaps in preventive behaviors (4, 5). In Poland, skin cancers constituted approximately 8% of all cancers diagnosed in 2021, with melanoma accounting for 11.9 and 10.8 cases per 100,000 in men and women, respectively, while other types of skin cancers were reported at 50.2 and 32.8 cases per 100,000. These figures highlight the pressing need to address skin cancer prevention (6). However, in Poland, while national cancer prevention campaigns address various malignancies, skin cancer often receives less focused attention, highlighting the need for tailored educational interventions, particularly among younger populations. Early detection and prevention are critical for reducing the burden of both melanoma and non-melanoma skin cancers (NMSC), as they are largely treatable when identified at an early stage (7–9).

Thus, public education and awareness campaigns are emphasized as key strategies in combating this trend, yet gaps in knowledge and behaviors persist, even among those in the medical field (10). Medical students are uniquely positioned as future healthcare providers to influence public awareness and encourage preventive behaviors, including skin self-examinations and sun protection measures (11). However, several studies have shown variability in their own understanding and practices regarding skin cancer prevention (12, 13). Similarly, non-medical students represent a diverse segment of young adults who are equally at risk of developing skin cancer due to inadequate awareness and behaviors, such as insufficient use of sunscreen and prolonged exposure to ultraviolet (UV) radiation (14–16). Understanding the attitudes and behaviors of non-medical students is equally critical, as this group represents a diverse population that may be overlooked in traditional health promotion strategies.

Comparative assessments of attitudes and behaviors between medical and non-medical students can provide valuable insights into the effectiveness of current educational initiatives and highlight areas for improvement. Previous research has suggested that while medical students may possess greater baseline knowledge, this does not always translate into better preventive practices, underscoring the need for targeted interventions within this population (17). Furthermore, understanding the attitudes of non-medical students is essential to designing inclusive public health strategies that resonate with broader demographics (18).

Although numerous studies emphasize the public health importance of skin cancer, a persistent gap exists in understanding which factors truly drive preventive behaviors among young adults. Comparing medical and non-medical students is particularly relevant because medical students as future healthcare providers – are expected to model appropriate sun-protective behaviors and to educate the public on skin cancer prevention. Their attitudes and practices therefore serve as an important benchmark for evaluating whether current medical curricula adequately prepare students for this role. Previous studies have suggested that medical students tend to exhibit higher knowledge levels and more favorable sun-protective behaviors than their non-medical peers; however, these findings may be confounded by demographic factors, especially gender, since women consistently report higher sunscreen use and greater awareness of skin cancer risks regardless of academic field (19–21).

This study addresses a critical gap in the literature by controlling for gender in comparative analyses, allowing us to determine whether differences attributed to medical training persist after adjusting for demographic composition. Our findings reveal that the apparent advantage of medical students in sunscreen adherence and knowledge is entirely explained by the higher proportion of women in the medical student group, rather than by their academic background itself. When gender was included in multivariable models, field of study no longer predicted sunscreen use or knowledge. These results provide important clarification for the interpretation of prior comparative studies, many of which did not sufficiently account for gender distribution. By demonstrating that gender – not academic discipline – is the key determinant of sun-protective behaviors, our study highlights the need for targeted interventions, particularly those aimed at improving sun protection among male students across all fields of study. This methodological approach fills a meaningful gap in existing research and offers a more accurate foundation for designing effective educational and public health strategies.This study aims to evaluate and compare the behaviors, attitudes, and awareness regarding skin cancer among medical and non-medical students. By identifying specific gaps and differences, the findings can guide the development of tailored educational programs to enhance skin cancer prevention efforts across various populations.

## 2 Methods

This study employed a cross-sectional design to assess behaviors, attitudes, and awareness regarding skin cancer among medical and non-medical students in Poland. Data were collected using an online survey distributed via Google Forms, which allowed for anonymous participation and broad accessibility.

### 2.1 Participants

The survey was completed by 411 students, comprising medical and non-medical students from various universities across Poland. Among the respondents, 80.8% identified as female, and 19.2% as male. The participants ranged in age, with the majority being between 21 and 28 years old. Inclusion criteria required participants to be currently enrolled students, with no restrictions on age, gender, or academic year. The survey link was distributed through university mailing lists, student social media platforms, and online academic forums to ensure broad reach and accessibility.

### 2.2 Instrument

The survey questionnaire was developed specifically for this study, based on prior validated instruments and adapted to address the research objectives. It included three main sections:

Demographics Age, gender, university type (medical or non-medical), and year of study;

Knowledge and Awareness: Questions on skin cancer risk factors, symptoms, and prevention strategies; Attitudes and Behaviors: Assessment of attitudes toward sun protection, frequency of sunscreen use, and engagement in preventive measures such as skin self-examinations. The questionnaire consisted of 36 closed-ended questions with multiple-choice and Likert scale responses to facilitate quantitative analysis (**Supplementary File 1**).

### 2.3 Data collection

Data were collected between December 2023, and October 2024, through the Google Forms platform. Participation was voluntary, and informed consent was obtained electronically at the beginning of the survey.

### 2.4 Data analysis

Data analysis was conducted using Python, employing statistical libraries such as scipy, statsmodels, and pandas to ensure robust and reproducible results. Descriptive statistics were used to summarize participant demographics, knowledge levels, and sun-protective behaviors, with results expressed as means, standard deviations, frequencies, and proportions. Chi-squared tests were performed to assess associations between categorical variables, such as gender and sunscreen use; Fisher’s exact test was used in place of chi-squared when any expected cell frequency was below 5. The phi coefficient (φ) was reported as a measure of effect size for statistically significant 2x2 associations. To control for multiple comparisons across the 29 tests in **Table 1**, the Benjamini–Hochberg false discovery rate (FDR) correction was applied at α = .05. Shapiro–Wilk tests indicated that all composite scores deviated significantly from normality (all *p* <.001); therefore, Mann–Whitney U tests were used for between-group comparisons of composite scores, and Spearman’s rank correlation coefficients (*rs*) were calculated)to evaluate non-linear relationships between variables. Model fit was assessed using the F-statistic (*F*) and the coefficient of determination (*R²*). To identify predictors of sunscreen use, a multivariable linear regression model was developed, including gender, age group, field of study, and knowledge score as covariates. Model fit was assessed using F-statistics and adjusted R-squared values, with statistical significance defined as p<0.05. Key variables included sunscreen use scores, derived from questions on sunscreen use across varying conditions, and sun avoidance scores, based on behaviors such as seeking shade and avoiding peak sun hours. Knowledge scores were calculated by aggregating responses to questions assessing awareness of skin cancer risk factors, symptoms, self-examination techniques, and preventive practices. All statistical analyses were conducted on a cleaned dataset, excluding cases with missing or invalid responses. Data visualization was performed using matplotlib and seaborn, with graphical representation of trends and group comparisons to enhance interpretability. The analysis emphasized the identification of disparities in sun-protective behaviors and the role of knowledge as a determinant of sunscreen use, providing a basis for targeted interventions in skin cancer prevention.

**Table 1:** Comparison of sun protection behaviors, knowledge, and risk factors for skin cancer between male and female respondents.

History and family risk factors			
Question	Male yes (%)	Female yes (%)	P-value*
Have you ever been diagnosed with skin cancer?	0.00%	1.22%	0.737**
Has anyone in your family had skin cancer?	14.29%	14.37%	1.000
Did you often experience sunburns during childhood?	29.87%	28.13%	0.870
Do you have blue eyes?	44.16%	35.17%	0.180
Do you have blonde or red hair?	35.06%	39.76%	0.529
Sun Protection Behaviors			
**Question**	**Male Yes (%)**	**Female Yes (%)**	p-value*
Do you use sunscreen on sunny days?	61.04%	84.71%	**<.001**
Do you use sunscreen when spending time at the beach?	92.21%	95.11%	0.466
Do you use sunscreen on cloudy days?	16.88%	51.68%	**<.001**
Do you use sunscreen daily?	12.99%	44.95%	**<.001**
Do you reapply sunscreen throughout the day?	28.57%	23.55%	0.438
Do you avoid the sun and stay in the shade on sunny days?	66.23%	61.16%	0.487
Do you wear a hat on sunny days?	54.55%	42.51%	0.074
Do you use tanning beds?	1.30%	7.03%	0.099**
Do you sunbathe during vacations?	33.77%	55.35%	**0.001**
Do you go outside between 10:00 AM and 4:00 PM without sun protection?	79.22%	67.58%	0.062
Attitudes and Barriers to Sun Protection			
**Question**	**Male Yes (%)**	**Female Yes (%)**	p-value*
I believe sun protection is important.	97.40%	98.17%	1.000**
I sometimes forget to use sunscreen.	84.42%	78.29%	0.298
I don’t have enough time during the day to apply sunscreen.	46.75%	28.75%	**0.004**
Sun protection is too expensive.	24.68%	25.99%	0.926
I use sunscreen to prevent signs of aging, not because I’m afraid of skin cancer.	18.18%	41.28%	**<.001**
Skin Monitoring			
**Question**	**Male Yes (%)**	**Female Yes (%)**	p-value*
Have you ever undergone a dermatoscopic skin examination?	45.45%	44.34%	0.961
Do you regularly observe your skin and monitor any changes?	79.22%	81.65%	0.742
Knowledge About Skin Cancer			
**Question**	**Male Yes (%)**	**Female Yes (%)**	p-value*
Do you think you have sufficient knowledge about skin cancer?	62.34%	60.55%	0.873
Do you have knowledge about the main risk factors for skin cancer?	85.71%	83.49%	0.759
Do you have any knowledge about self-examining your skin?	71.43%	66.97%	0.537
Can chronic skin irritation lead to skin cancer?	84.42%	73.09%	0.054
Do you know the most common areas where melanoma occurs on the body?	45.45%	42.81%	0.770
Do you know other methods of diagnosing melanoma besides visual assessment of skin lesions?	61.04%	56.27%	0.527
Do you know the characteristics of skin cancer?	75.32%	70.03%	0.434

*p-value calculated with chi2 test; **p-value calculated with Fisher’s exact test;.

Benjamini–Hochberg false discovery rate (FDR) correction was applied to control for multiple comparisons across the 29 tests in this table. All six statistically significant results (bolded) remained significant after FDR correction (adjusted p <.05).

Composite scores were calculated to quantify sun-protective behaviors and skin cancer–related knowledge. All scores were derived using a simple additive scoring approach based on binary responses to individual questionnaire items. Each item was coded as 1 point for a protective or correct response (“Yes”/”True”) and 0 points for a non-protective or incorrect response (“No”/”False”). For items where endorsement indicated a risk behavior (e.g., use of tanning beds, failure to avoid sun exposure), responses were reverse-coded prior to score calculation to ensure consistent directionality across all scales, with higher scores uniformly reflecting more favorable behaviors or higher knowledge.

Four domain-specific composite scores were constructed as follow. Sunscreen Use Score, based on six items assessing sunscreen application in different situations (sunny days, beach exposure, cloudy days, daily use, and reapplication), with a possible range of 0–6 points. Sun Avoidance Score, based on two items evaluating avoidance of direct sun exposure and staying in the shade during peak UV hours, with a possible range of 0–2 points. Other Protective Measures Score, based on three items assessing the use of additional protective strategies (e.g., wearing a hat, regular skin self-examination, avoidance of tanning beds), with a possible range of 0–3 points. Knowledge Score, based on eight items assessing awareness of skin cancer risk factors, symptoms, and diagnostic methods, with a possible range of 0–8 points. Higher scores indicated greater adherence to sun-protective behaviors or higher levels of skin cancer–related knowledge. This scoring strategy was selected to maximize transparency, interpretability, and comparability with prior questionnaire-based studies assessing preventive behaviors and cancer-related knowledge in student and young adult populations.

### 2.5 Ethics

The study was conducted in accordance with the principles of the Declaration of Helsinki. Ethical approval and informed consent were not required in accordance with national regulations on survey-based research that does not involve personal or sensitive data.

### 2.6 Limitations of the sampling method

This study utilized a convenience sampling approach based on voluntary participation in an online survey distributed through university mailing lists, social media platforms, and student forums. Although this method enabled broad reach and efficient data collection, it introduces several limitations. First, the reliance on self-selection may have created selection bias, as students with greater interest in health topics or personal experience with sun protection behaviors may have been more likely to participate. Second, the gender distribution in our sample was markedly imbalanced, with women representing over 80% of respondents, which may limit the generalizability of findings to male students. Third, because the survey was disseminated online, participation was restricted to individuals with internet access and active engagement on university or social platforms. Finally, the use of non-probabilistic sampling means the results should not be interpreted as nationally representative but rather reflective of trends within a self-selected group of Polish university students.

## 3 Results

### 3.1 Demographic characteristics of students

The survey was completed by 411 respondents; however, 404 questionnaires were correctly completed and met the inclusion criteria, and therefore were included in the final analysis. The study included 404 participants, with the majority being female (80.94%, 327 participants) compared to male respondents (19.06%, 77 participants). Participants were categorized into four age groups: 18–20 years (11.63%, 47 participants), 21–23 years (46.29%, 187 participants), 24–26 years (29.46%, 119 participants), and those older than 26 years (12.38%, 50 participants).

Regarding fields of study, 58.66% of participants (237 participants) were enrolled in medical fields, while 41.34% (167 participants) were from non-medical disciplines. The distribution across academic years was as follows: first year (5.94%, 24 participants), second year (18.32%, 74 participants), third year (19.80%, 80 participants), fourth year (14.60%, 59 participants), fifth year (31.19%, 126 participants), and sixth year (10.15%, 41 participants).

Sunscreen use was reported by 80.20% of participants (324 participants), while 19.80% (80 participants) indicated they did not use sunscreen. Sunscreen use reported in **Table 2** reflects self-reported ever-use on sunny days, assessed using a binary (yes/no) response. This measure does not indicate frequency or regularity of sunscreen application.

**Table 2:** Demographic characteristics of students.

Characteristic	Category	Count (%)
Gender	Male	77 (19.06%)
	Female	327 (80.94%)
Age group	18–20	47 (11.63%)
	21–23	187 (46.29%)
	24–26	119 (29.46%)
	>26	50 (12.38%)
Field of study	Medical	237 (58.66%)
	Non-medical	167 (41.34%)
Year of study	1st year	24 (5.94%)
	2nd year	74 (18.32%)
	3rd year	80 (19.80%)
	4th year	59 (14.60%)
	5th year	126 (31.19%)
	6th year	41 (10.15%)
Sunscreen use	Yes	324 (80.20%)
	No	80 (19.80%)

### 3.2 Sun protection behaviors, knowledge, and risk factors for skin cancer among male and female students

A survey was conducted to assess sun protection behaviors, knowledge, and risk factors for skin cancer among men and women (**Table 1**).

#### 3.2.1 History and family risk factors

None of the men and 1.22% of the women reported being diagnosed with skin cancer, a difference that was not statistically significant (*p* = .737) (**Table 1**). Similarly, no significant association was observed between gender and having a family history of skin cancer, with 14.29% of men and 14.37% of women reporting such a history (*p* = 1.000. Frequent sunburns during childhood were reported by 29.87% of men and 28.13% of women, a non-significant difference (*p* = .870). Among phenotypic traits, 44.16% of men and 35.17% of women reported having blue eyes (*p* = .180), while 35.06% of men and 39.76% of women reported having blonde or red hair (*p* = .529).

#### 3.2.2 Sun protection behaviors

Women were significantly more likely to use sunscreen on sunny days (84.71%, see **Table 1**) compared to men (61.04%) (*χ2*(1, *N* = 404)=33.18,*p* <.001,ϕ=.29). The odds of women using sunscreen were 3.57 times higher than men (OR = 3.57,95%CI[2.20,5.80]). Sunscreen use at the beach was reported by 92.21% of men and 95.11% of women (p=.466). However, on cloudy days, 51.68% of women used sunscreen compared to only 16.88% of men (*χ2*(1, *N* = 404)=33.57, *p* <.001;OR=5.33,95%CI[2.77,10.28]). Daily sunscreen use was reported by 44.95% of women and 12.99% of men (*χ2*(1, *N* = 404)=42.87, *p* <.001; OR = 5.30,95%CI[2.82,9.95]). Reapplication of sunscreen throughout the day was reported by 28.57% of men and 23.55% of women (*p* = .438).

In addition, the mean sunscreen use scores were significantly higher for women (3.24, *SD* = 1.66) compared to men (2.40, *SD* = 1.57) (*U=8072.0*, *p* <.001)., further supporting the finding that women were more likely to engage in sunscreen use behaviors than men.

Avoiding the sun and staying in the shade was reported by 66.23% of men and 61.16% of women (*p* = .487). Wearing hats on sunny days was reported by 54.55% of men and 42.51% of women, a difference that approached but did not reach statistical significance (*p* = .074). Tanning bed use was reported by 1.30% of men and 7.03% of women (*p* = .099). Sunbathing during vacations was significantly more common among women (55.35%) than men (33.77%) (*χ2*(1, *N* = 404)=10.73,*p* = .00;1 OR = 2.44, 95%CI[1.44,4.14]). Going outside between 10:00 AM and 4:00 PM without sun protection was reported by 79.22% of men and 67.58% of women (*p* = .062).

For sun avoidance behaviors, the mean scores for women (0.94, *SD* = 0.73) were slightly higher than those for men (0.87, *SD* = 0.61), but this difference was not statistically significant (*t*(402)=−0.73, *p* = .468).

For other protective measures, men had marginally higher scores (2.32, *SD* = 0.66) than women (2.17, *SD* = 0.71), but this difference was not statistically significant (*t*(402)=1.72, *p* = .086).

#### 3.2.3 Attitudes and barriers to sun protection

Most participants agreed on the importance of sun protection, with 97.40% of men and 98.17% of women endorsing its importance (*p* = 1.000, see **Table 1**). Forgetting to use sunscreen was reported by 84.42% of men and 78.29% of women (*p* = .298). Time constraints as a barrier to sunscreen use were significantly more common among men (46.75%) than women (28.75%) (*χ2*(1, *N* = 404)=8.47, *p* = .004; OR = 2.18, 95%CI[1.31,3.62]). Women were more likely to report using sunscreen to prevent signs of aging rather than for cancer prevention (41.28% vs. 18.18%) (*χ2*(1, *N* = 404)=22.04,*p* <.001; OR = 3.24, 95%CI[1.69,6.23]). The perception that sunscreen is too expensive was reported by 24.68% of men and 25.99% of women (*p* = .926).

#### 3.2.4 Skin monitoring

Undergoing a dermatoscopic skin examination was reported by 45.45% of men and 44.34% of women (*p* = .961, see **Table 1**). Regular observation of the skin and monitoring for changes was reported by 79.22% of men and 81.65% of women (*p* = .742).

#### 3.2.5 Knowledge about skin cancer

62.34% of men and 60.55% of women believed they had sufficient knowledge about skin cancer (*p* = .873, see **Table 1**). Awareness of the main risk factors was reported by 85.71% of men and 83.49% of women (*p* = .759). Knowledge of self-examination techniques was reported by 71.43% of men and 66.97% of women (*p* = .537). Awareness of the potential for chronic skin irritation to cause skin cancer was higher in men (84.42%) than in women (73.09%), although the difference was not statistically significant (*p* = .054). Knowledge of common melanoma sites was reported by 45.45% of men and 42.81% of women (*p* = .770), and awareness of other diagnostic methods besides visual assessment was comparable (61.04% of men vs. 56.27% of women,*p* = .527). Lastly, knowledge of the characteristics of skin cancer was reported by 75.32% of men and 70.03% of women (*p* = .434).

### 3.3 Comparison of sun-protection behaviors between medical and non-medical fields

Given the potential influence of educational background on health-related behaviors, this section examines differences in sun-protection practices between participants from medical and non-medical fields. **Table 3** provides an overview of the mean scores, standard deviations, and statistical tests assessing sunscreen use, sun avoidance, and other protective measures by field of study.

**Table 3:** Summary scores of sun-protection behaviors among medical and non-medical students.

Category	Non-Medical, mean (SD)	Medical, mean (SD)	U-statistic	p-value**
Sunscreen Use	2.75 (1.63)	3.31 (1.67)	16615.5	0.005
Sun Avoidance	0.83 (0.69)	0.99 (0.72)	17362.0	0.023
Other Measures	2.15 (0.71)	2.24 (0.70)	18479.5	0.216

**Test performed with Mann-Whitney U testParticipants from medical fields reported significantly higher sunscreen use scores (*M* = 3.31, *SD* = 1.67) compared to participants from non-medical fields (*M* = 2.75, *SD* = 1.63), *U=16615.5*, *p* = .005. Sun avoidance scores were slightly higher among participants from medical fields (*M* = 0.99, *SD* = 0.72) compared to non-medical fields (*M* = 0.83, *SD* = 0.69). This difference was statistically significant (*t(*402*)*=−2.31, *p* = .022).

Participants from medical fields also had slightly higher scores for other protective measures *(M* = 2.24, *SD* = 0.70) compared to non-medical fields (*M* = 2.15, *SD* = 0.71). This difference was not statistically significant (*t(*402*)*=−1.21*, p* = .225).

### 3.4 Influence of gender and family history on skin cancer knowledge

To determine whether knowledge about skin cancer is influenced by gender or a personal and/or family history of skin cancer, we analyzed participants’ knowledge scores and assessed potential differences across these factors (**Table 4**).

**Table 4:** Comparison of knowledge scores between study groups according to gender, personal and/or family history, and field of study (Mann–Whitney U test).

Comparison group	Mean ± SD (Group 1)	Mean ± SD (Group 2)	U-statistic	p-value
Overall sample	5.41 ± 2.30	—	—	—
Male vs Female	5.65 ± 2.36	5.35 ± 2.29	13753.5	0.201
Personal and/or family history (Yes vs No)	5.80 ± 2.10	5.34 ± 2.33	9327.5	0.172
Medical vs Non-medical field of study	6.23 ± 2.07	4.23 ± 2.33	29321.5	<0.001

The overall mean knowledge score across all participants was 5.41 (*SD* = 2.30).

The mean knowledge score for men was 5.35 (*SD* = 2.29), while for women, it was slightly higher at 5.65 (*SD* = 2.36). However, this difference was not statistically significant (U = 13753.5, *p* = .201).

Participants with a personal or family history of skin cancer had a mean knowledge score of 5.80 (*SD* = 2.10), compared to 5.34 (*SD* = 2.33) for those without such a history. This difference was not statistically significant (U = 9327.5, *p* = .172).

Participants from medical fields had significantly higher knowledge scores (**Table 4**) (*M* = 6.23, *SD* = 2.07 compared to those from non-medical fields (*M* = 4.23, *SD* = 2.33). This difference was statistically significant, U = 29321.5,*p* <.001. The results indicate that individuals in medical fields possess greater knowledge about skin cancer than their counterparts in non-medical fields.

### 3.5 Relationship between knowledge and sun-protection behaviors

**Figure 1:**
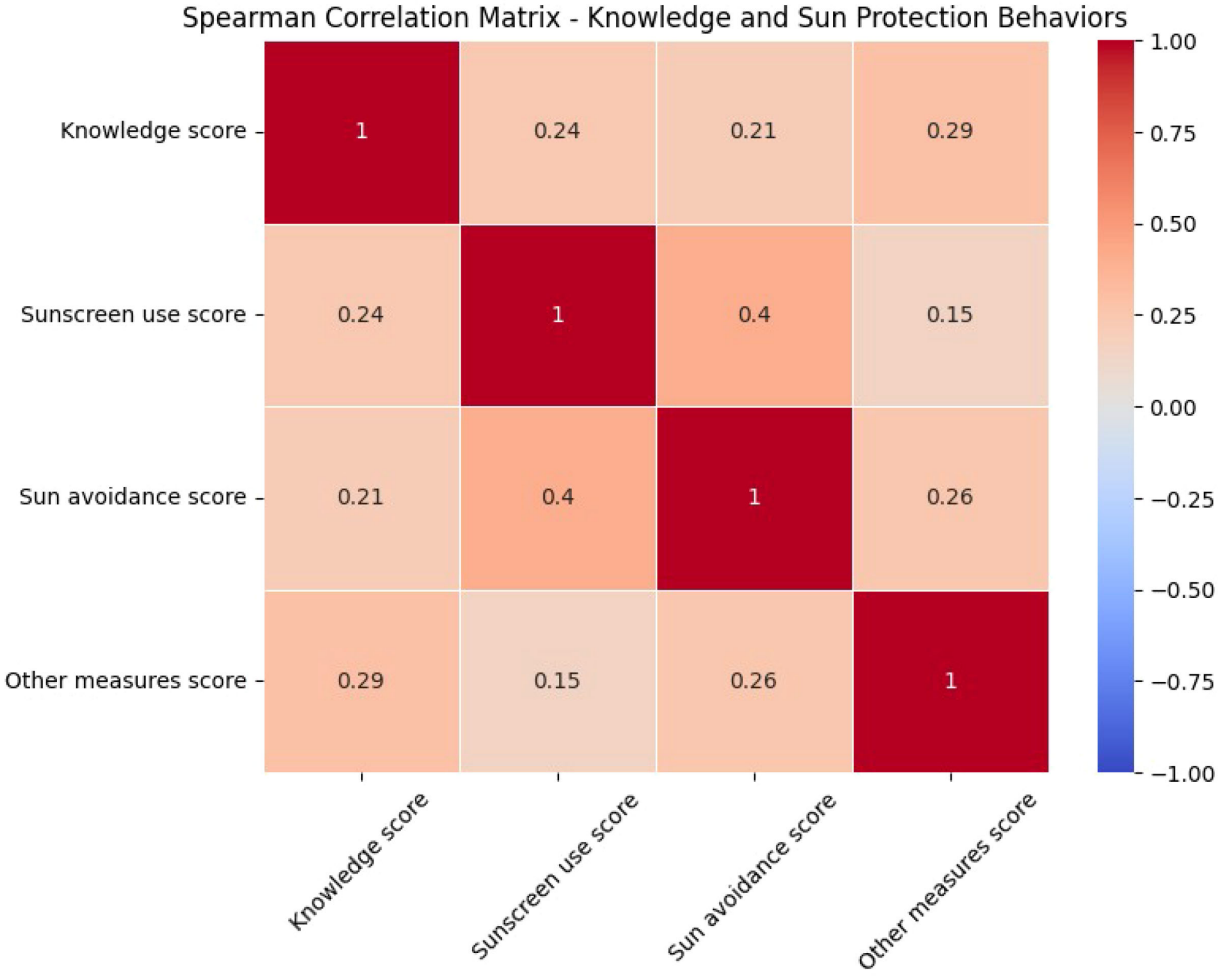
Spearman correlation matrix of knowledge and sun protection behaviors.

A Spearman correlation analysis was performed to examine the relationships between knowledge scores and sun protection behaviors, including sunscreen use, sun avoidance, and other protective measures (**Figure 1**).

Knowledge scores were weakly correlated with sunscreen use (*r_s_* = .24), indicating that individuals with higher knowledge were slightly more likely to use sunscreen. A similarly weak correlation was observed between knowledge scores and sun avoidance behaviors (*r_s_* = .21), suggesting a limited association between knowledge and avoiding sun exposure.

The strongest correlation involving knowledge was with other protective measures (*r_s_* = .29), highlighting that individuals with higher knowledge scores were more likely to engage in additional protective practices, such as wearing hats or avoiding tanning beds. Sunscreen use and sun avoidance behaviors were moderately correlated (*r_s_* = .40), suggesting that these behaviors often co-occurred among participants. A weak correlation was observed between sunscreen use and other protective measures (*r_s_* = .15), reflecting limited overlap between these behaviors.

Finally, a moderate correlation between sun avoidance and other protective measures (*r_s_* = .26) suggested that individuals who avoided sun exposure were also more likely to engage in other protective behaviors. Overall, the findings suggest that knowledge about skin cancer is positively associated with sun protection behaviors, with the strongest relationships observed for additional protective measures.

In addition, a Mann–Whitney U test comparing knowledge scores between self-monitoring and non-self-monitoring groups revealed a significant difference *(U* = 20419.0*, p* <.001, rank-biserial r=-0.64). Participants who regularly monitored their skin (self-monitoring group) scored significantly higher (*M* = 5.70, *SD* = 2.04) than those who did not (*M=*3.17,*SD* = 2.12). These findings suggest that proactive self-care behaviors are associated with better knowledge about skin cancer risks.

Moreover, participants who reported positive attitudes toward sun protection were significantly more likely to use sunscreen (χ2 = 13.05, p<.001,φ=.18). Similarly, forgetfulness was identified as a key barrier to sunscreen use (χ2 = 13.56, p<.001,φ=.18), with individuals who reported frequent forgetfulness demonstrating lower adherence to sun-protection behaviors. Perceived time constraints were also significantly associated with reduced sunscreen use (χ2 = 6.07, p=.014,φ=.08).

Overall, the findings suggest that knowledge about skin cancer is positively associated with sun protection behaviors, with the strongest relationships observed for additional protective measures. Additionally, proactive self-monitoring and addressing barriers such as forgetfulness and time constraints may further enhance adherence to sun-protective practices.

### 3.6 Age and gender trends in sun-protection behaviors

**Figure 2:**
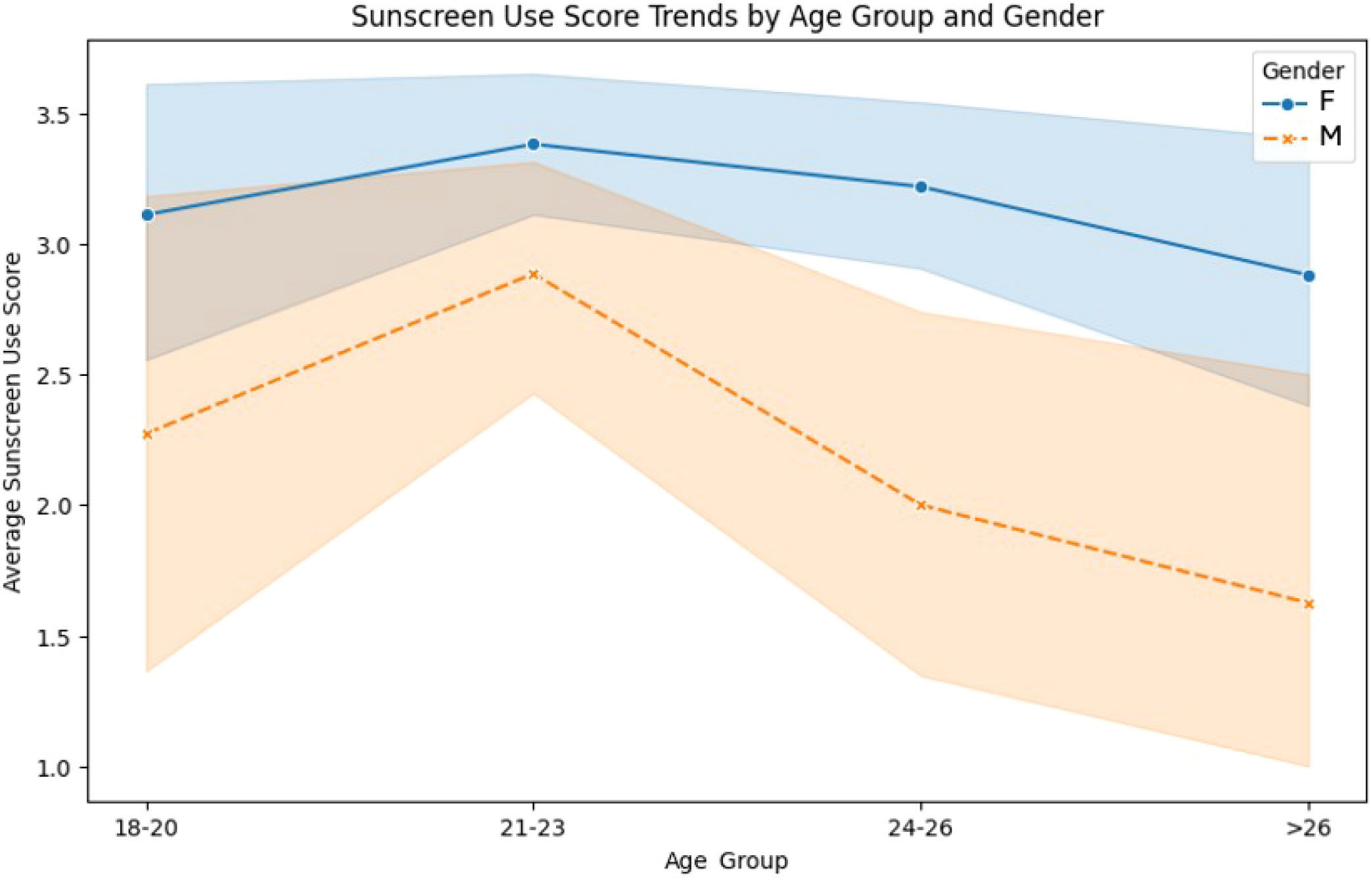
Sunscreen use score trends by age group and gender.

The mean sunscreen use scores were higher among women across all age groups compared to men (**Figure 2**). The highest sunscreen use score for both genders was observed in the 21–23 age group, with women averaging above 3.5 and men slightly below 3.0.

A noticeable decline in sunscreen use was observed for both genders in the 24–26 and >26 age groups. Men exhibited a sharper decrease, with scores dropping below 2.0 in the >26 age group.

**Figure 3:**
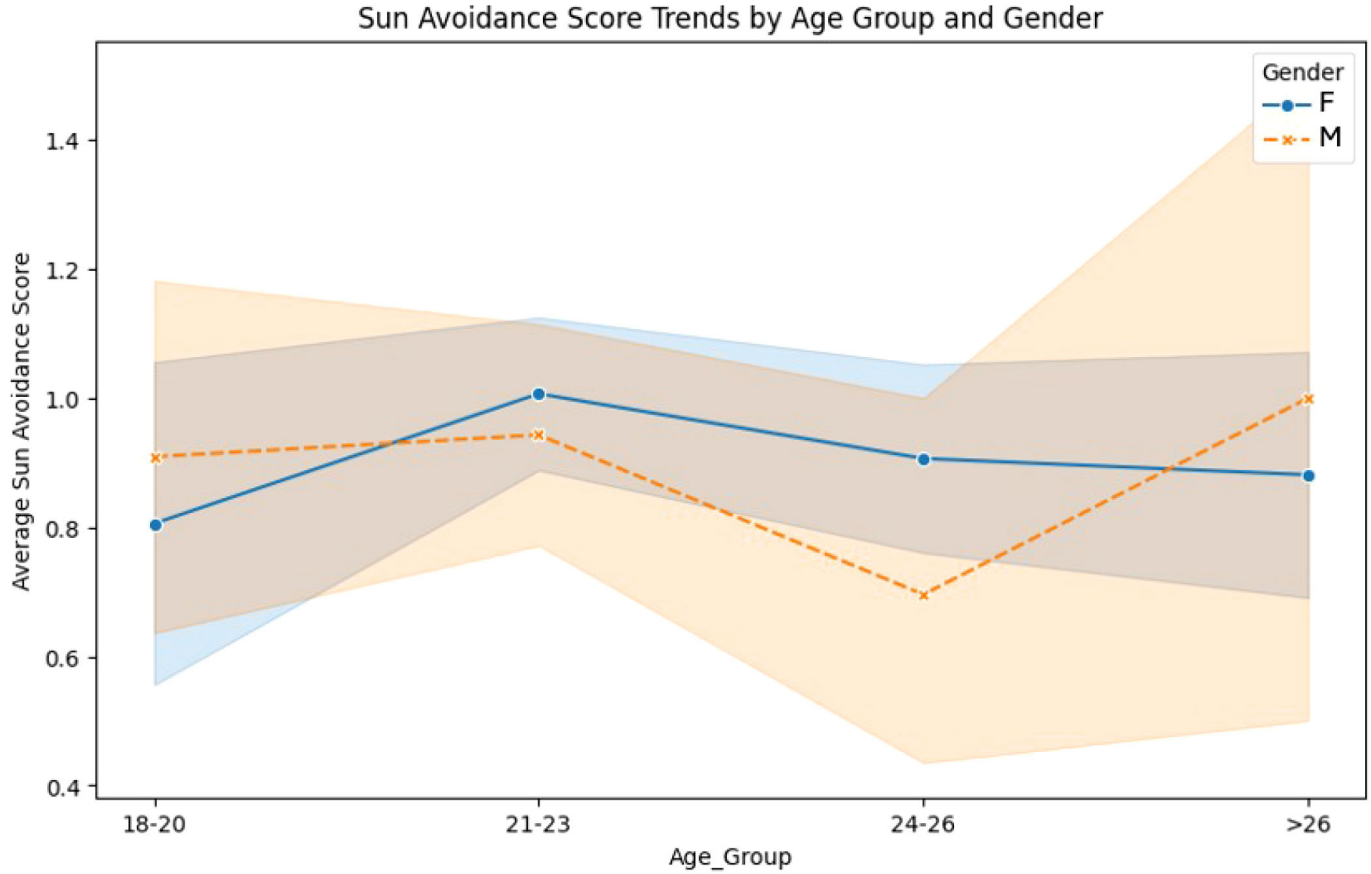
Sun avoidance score trends by age group and gender.

For women, the sun avoidance score remained relatively stable across age groups (**Figure 3**), hovering around 1.0. Men’s sun avoidance scores were lower than women’s in most age groups but showed an increase in the >26 group, where men surpassed women. The difference in trends highlights an interaction effect where older men tend to adopt sun avoidance measures more frequently.

**Figure 4:**
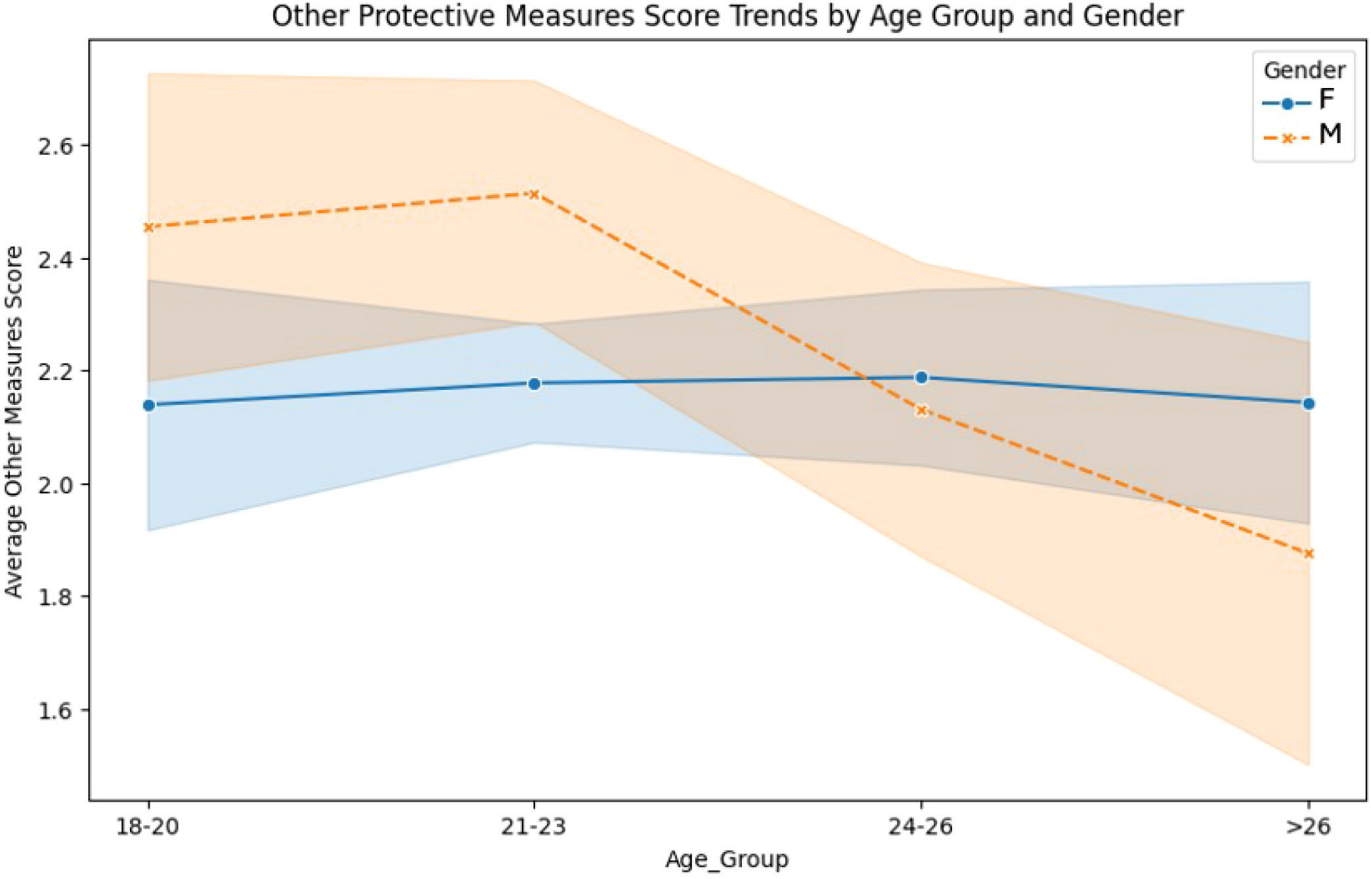
Other protective measures score trends by age group and gender.

Men had consistently higher scores for other protective measures than women across most age groups (**Figure 4**). While women’s scores remained relatively stable around 2.1, men showed a gradual decline from 2.5 in the 18–20 age group to approximately 1.8 in the >26 group. The discrepancy suggests that younger men are more likely to engage in additional protective measures, but this behavior diminishes with age. Detailed mean values and standard deviations for sunscreen use, sun avoidance, and other protective measures stratified by age group and gender are presented in **Table 5**.

**Table 5:** Sun protection behavior scores by age group and gender.

Age group	Gender	Sunscreen use score mean	Sunscreen use score Std	Sun avoidance score mean	Sun avoidance score Std	Other measures score mean	Other measures score Std
18-20	Female	3.11	1.62	0.81	0.75	2.14	0.72
	Male	2.27	1.68	0.91	0.54	2.45	0.52
21-23	Female	3.38	1.67	1.01	0.75	2.18	0.66
	Male	2.89	1.39	0.94	0.54	2.51	0.66
24-26	Female	3.22	1.64	0.91	0.73	2.19	0.80
	Male	2.00	1.76	0.70	0.70	2.13	0.63
>26	Female	2.88	1.68	0.88	0.67	2.14	0.72
	Male	1.63	1.19	1.00	0.76	1.88	0.64

An Ordinary Least Squares (OLS) regression was performed to assess the impact of gender, age group, field of study (medical vs. non-medical), and knowledge score on sunscreen use scores. The model was statistically significant (*F*(6,396)=9.491,*p* <.001), explaining 12.6% of the variance in sunscreen use scores (*R^2^* = .126, adjusted *R^2^* = .112).

The intercept was *β* = 2.3638 (*p* <.001), representing the baseline sunscreen use score for the reference categories (male, age 18-20, non-medical field, and knowledge score of zero). Gender was a significant predictor (*β*=−0.9148, *p* <.001), with men scoring 0.91 points lower on average than women on sunscreen use. None of the age group comparisons were statistically significant: Age 21-23 (*β* = 0.2725, *p* = .291); Age 24-26 (*β* = 0.0111, *p* = .967); Age >26 (*β*=−0.2617, *p* = .415) Field of study (medical vs. non-medical) approached statistical significance (*β*=−0.2480, *p* = .103). Participants from non-medical fields tended to have lower sunscreen use scores. Knowledge score was a statistically significant predictor (*β* = 0.1654, *p* <.001). For every one-point increase in knowledge score, sunscreen use increased by 0.165 points on average.

Thus, the results indicate that gender and knowledge score are the primary predictors of sunscreen use, with women and participants possessing higher knowledge about skin cancer demonstrating greater adherence to sunscreen use. While the medical field showed a marginal effect, it did not reach statistical significance. Age group had no significant impact on sunscreen use scores.

### 3.7 Predictors of sunscreen use among polish medical and non-medical students

**Figure 5:**
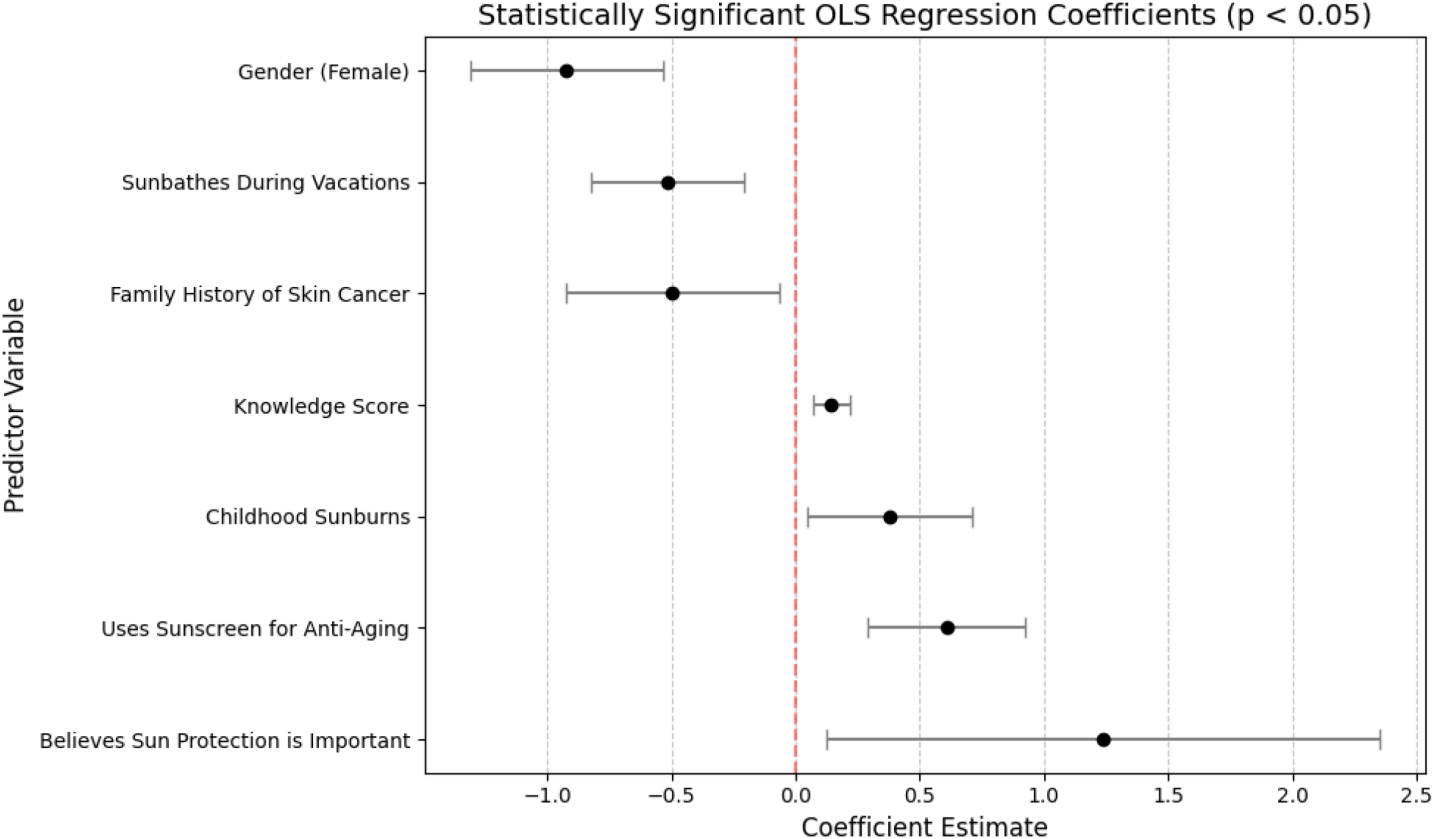
Statistically significant OLS regression coefficients (*p* < 0.05) predicting sunscreen use among Polish students. The plot displays coefficient estimates with 95% confidence intervals for key predictors. Negative coefficients (e.g., male gender, sunbathing during vacations, family history of skin cancer) indicate reduced sunscreen use, while positive coefficients (e.g., higher knowledge scores, childhood sunburns, belief in the importance of sun protection, use of sunscreen for anti-aging) are associated with increased sunscreen adherence. The red dashed line represents the null effect (*β* = 0).

An OLS regression model (*R²* = 0.228, *F*(15, 388) = 7.64, *p* < .001) revealed several predictors of sunscreen use (**Figure 5**). Males used sunscreen less frequently than females (*b* = –0.92, *SE* = 0.20, *t* = –4.66, *p* < .001), whereas higher knowledge scores (*b* = 0.14, *SE* = 0.04, *t* = 3.84, *p* < .001) and a positive attitude toward sun protection (*b* = 1.24, *SE* = 0.57, *t* = 2.18, *p* = .030) were associated with increased use. Habitual sunbathing during vacations predicted lower sunscreen use (*b* = –0.52, *SE* = 0.16, *t* = –3.28, *p* = .001), while frequent childhood sunburns were linked to higher use (*b* = 0.38, *SE* = 0.17, *t* = 2.28, *p* = .023). Notably, a family history of skin cancer was associated with reduced sunscreen use (*b* = –0.50, *SE* = 0.22, *t* = –2.28, *p* = .023), although this finding should be interpreted cautiously given that only four participants reported a family history of skin cancer. Field of study did not significantly affect sunscreen practices (*b* = –0.17, *SE* = 0.17, *t* = –1.02, *p* = .311).

## 4 Discussion

Skin cancer represents a significant and growing public health challenge, with both melanoma and non-melanoma skin cancers (NMSC) contributing to increasing morbidity and healthcare costs worldwide (22). Despite widespread prevention efforts, disparities in knowledge, behaviors, and systemic accessibility persist, as evidenced by our findings and corroborated by global literature (23, 24). Moreover, despite the advent of advanced diagnostic techniques and innovative therapies, the incidence and, in certain populations, mortality rates of skin cancers continue to rise globally, underscoring the need for comprehensive strategies that integrate early detection, effective treatment, and robust public health interventions (25–31).

Our study underscores notable differences in knowledge and preventive behaviors between medical and non-medical students, reflecting the influence of educational background. Medical students demonstrated higher awareness of skin cancer risk factors and prevention strategies, aligning with research emphasizing the role of specialized education in fostering health literacy. However, a gap persisted between knowledge and practice, as reflected by suboptimal sunscreen use even among medical students. This pattern mirrors broader evidence that knowledge frequently fails to translate into consistent sun-protective behaviors in young adults and student populations (32, 33).

In parallel, non-medical students exhibited lower adherence to protective measures such as sunscreen application and sun avoidance, despite acknowledging the importance of sun protection. This aligns with global data suggesting that young adults often underestimate ultraviolet (UV) risks in the context of cultural attitudes favoring tanned skin as a marker of health and attractiveness (34–36). Taken together, our results reinforce that increasing knowledge is necessary but insufficient; interventions should be explicitly designed to convert awareness into routine behavior, using approaches that address both cultural norms and practical implementation barriers (32, 33).

Consistent with the literature, female students in our sample reported higher adherence to sunscreen use and other protective measures than male students (18, 37, 38). This divergence is commonly linked to gendered perceptions of appearance, where women may prioritize sun protection to prevent photoaging rather than skin cancer per se (39–41). Conversely, men’s lower engagement in sun-protective behaviors is clinically important, given evidence linking lower preventive uptake and delayed health-seeking with a higher likelihood of advanced-stage melanoma at diagnosis (33). Interestingly, while men reported slightly better adherence to certain physical protective measures such as wearing hats, their overall lower sunscreen use reflects broader trends documented internationally and in studies focused on men’s skincare behaviors (42). These findings support the need for gender-tailored public health messaging that resonates with male audiences and addresses common barriers (e.g., perceived inconvenience, time constraints), while framing benefits in both health-related and appearance-related terms where appropriate (39–42).

Our findings align with international studies assessing behaviors, attitudes, and awareness regarding skin cancer among medical and non-medical students. Evidence from multiple countries indicates that medical students generally demonstrate higher knowledge and awareness than non-medical students, yet this advantage does not consistently translate into higher adherence to sun-protective behaviors. For example, a cross-sectional study among medical students in Saudi Arabia reported high awareness of sun-exposure risks but limited regular sunscreen use, with substantially better adherence among females than males (43). Similarly, research from Iran found that although medical students outperformed non-medical students in knowledge and attitudes, preventive behaviors remained only moderate, underscoring the need to expand educational approaches beyond medical curricula (44). Comparable observations were reported in a multi-country survey including Poland, which showed higher recognition of cancer risk factors among medical students alongside persistent misconceptions and demographic variability, highlighting gaps in university-level health education (45). Studies from the United States further suggest that even students in health-related majors may continue to engage in high-risk behaviors, including intentional tanning and irregular sunscreen use (46, 47).

When placed in an international context, our results both confirm and refine patterns described in prior studies comparing medical and non-medical students. In our Polish sample, medical students initially appeared to have higher knowledge scores and more frequent sunscreen use than non-medical students; however, these differences disappeared after adjustment for gender. Female sex and higher knowledge scores emerged as the strongest predictors of sunscreen adherence, suggesting that demographic composition may explain apparent “field-of-study” effects in mixed student samples. This contrasts with earlier findings from Saudi Arabia and Iran, where medical students more consistently demonstrated superior knowledge, more favorable attitudes, and somewhat better preventive practices than their non-medical peers, although overall adherence still remained moderate (43, 44). In Saudi Arabia, for instance, avoidance of direct sunlight and other protective behaviors were common, yet regular sunscreen use remained limited—particularly among men (43). Similarly, Iranian medical students had higher knowledge and performance scores but still required strengthened educational interventions to improve practice (44).

Our findings are also consistent with multi-country evidence indicating that while medical or health-related education improves recognition of skin cancer risk factors, it does not necessarily translate into high compliance with sun-protective behaviors. International surveys across 23–32 countries show that medical students more often identify UV exposure, tanning beds, and sunbathing as carcinogenic; nonetheless, young adults, men, and individuals with lower education remain less likely to use sunscreen or seek shade (40, 48–50). Likewise, studies in France and the United States indicate that although health-related majors may display better knowledge, protective practices remain inconsistent, and regular skin self-examination is often reported by fewer than 40% (16, 47, 51).

Importantly, the interpretation of our findings should be framed within robust evidence that knowledge alone is a weak and inconsistent predictor of sustained sun-protective behavior. A systematic review demonstrated that while higher skin cancer knowledge is generally associated with greater awareness, its relationship with actual preventive behaviors is heterogeneous and inconsistent across outcomes (52). Broader syntheses similarly suggest that knowledge explains only a limited proportion of real-world sun-protective behavior and that effective interventions must address additional psychological and contextual determinants (53).

Consistent with health behavior frameworks, studies applying the Health Belief Model indicate that perceived barriers, self-efficacy, and perceived severity are often more influential determinants of sunscreen use than knowledge alone (54, 55). Meta-analytic evidence based on the Theory of Planned Behavior further shows that although attitudes, subjective norms, and perceived behavioral control explain a substantial share of sun-protective intentions, they account for a markedly smaller proportion of actual behavior—highlighting a persistent intention–behavior gap (56). Longitudinal and social-cognitive models also emphasize that sunscreen adherence is strongly shaped by habit formation, automaticity, and self-regulatory processes rather than information alone (57, 58). Population-based research using objective measures of UV exposure and sunburn outcomes suggests that routine protective patterns and consistent application behaviors may correlate more strongly with reduced sunburn risk than knowledge deficits per se (59–61). In practice, empirical evidence consistently identifies practical barriers—particularly forgetfulness and limited time—as key obstacles to regular sunscreen use among young adults, even when risk awareness is high (62–64). These frameworks provide a coherent explanation for our observation that knowledge correlated positively with protective behaviors but was insufficient to ensure adherence.

Poland’s healthcare infrastructure faces specific challenges in delivering effective skin cancer prevention (65). Only 25.4% of Poles declare having undergone a dermoscopic examination at least once in their lifetime (66). Limited access to affordable sunscreen and professional skin examinations may exacerbate disparities, especially among non-medical students, and reflects broader international evidence that socioeconomic conditions and structural access influence preventive uptake and downstream outcomes. Expanding access to subsidized prevention tools and professional screening, coupled with public awareness campaigns, could help reduce these gaps (65, 66).

Our results highlight the urgent need for targeted interventions to address lower sunscreen use among men and the broader knowledge–behavior gap across student populations. Integrating skin cancer prevention into general health promotion initiatives may improve awareness and foster protective habits across diverse demographic groups. Future research should investigate additional predictors of sun-protective behaviors, including environmental factors, phenotypic traits, and behavioral patterns, and should prioritize intervention studies that test the effectiveness of tailored educational campaigns and public health strategies in reducing skin cancer burden.

Finally, these findings should be interpreted cautiously in light of methodological research demonstrating that student samples may systematically differ from the general population, limiting external validity and generalizability of education-related differences in health behaviors (67–74). Because this study employed convenience sampling and voluntary participation, the sample may not fully represent the broader Polish student population. The overrepresentation of women further constrains the generalizability of gender-related conclusions, as observed disparities may differ in more balanced samples. Moreover, the absence of random sampling across multiple faculties and institutions restricts the external validity of our results. Future studies using stratified or random sampling across diverse academic disciplines would provide more robust evidence and allow stronger generalization of both field-of-study and gender-related differences in sun-protective behaviors.

## 5 Conclusions

In conclusion, this study demonstrates that gender—rather than field of study—is the primary determinant of sun-protective behaviors and skin cancer knowledge among Polish university students. Although medical students appeared to show more favorable behaviors and higher awareness in unadjusted analyses, these differences were explained by gender distribution rather than academic training. These findings underscore the limited generalizability of assumptions suggesting that medical students consistently outperform non-medical students in preventive behaviors.

Importantly, the results should not be extrapolated to all young adults or populations outside Poland. Instead, they highlight the need for targeted interventions, particularly for male students, who consistently exhibited lower adherence to sunscreen use and greater behavioral barriers. Future research should incorporate longitudinal designs, more diverse populations, and additional behavioral and psychological determinants to better understand the mechanisms underlying sun-protective behaviors in young adults.

## Data Availability

The raw data supporting the conclusions of this article will be made available by the authors, without undue reservation.
