# Supplementary material for: Assessment of behaviors, attitudes, and awareness regarding skin cancer among polish medical and non-medical students: comparative analysis
Source: Front Oncol. 2026 May 8;16:1697895. doi: 10.3389/fonc.2026.1697895 (PMC13193818; doi:10.3389/fonc.2026.1697895)
Supplement: Supplementary file 1 [file SupplementaryFile1.docx]

### **Survey on Behaviors, Attitudes, and Awareness of Skin Cancer Among Medical Students**

### **Questionnaire**

**Gender**

- Female
- Male

**Age:**

**University:**

**Field of Study:**

**Year of Study**

- 1
- 2
- 3
- 4
- 5
- 6

**Have you ever been diagnosed with skin cancer?**

- Yes
- No

**Has anyone in your family been diagnosed with skin cancer?**

- Yes, first-degree relatives
- Yes, second-degree relatives
- No

**How would you assess the number of moles on your body?**

- <10
- 11–50
- 51–100
- 100

**Have you ever undergone dermoscopic skin examination?**

- Yes
- No

**Do you have blue eyes?**

- Yes
- No

**Do you have blonde or red hair?**

- Yes
- No

**Did you frequently experience sunburns during childhood?**

- Yes
- No

### **Sun Protection Behaviors**

**Do you use sunscreen on sunny days?**

- Yes
- No

**Do you use sunscreen at the beach?**

- Yes
- No

**Do you use sunscreen on cloudy days?**

- Yes
- No

**Do you use sunscreen daily?**

- Yes
- No

**Do you reapply sunscreen during the day?**

- Yes
- No

**Do you avoid sun exposure and stay in the shade on sunny days?**

- Yes
- No

**Do you wear a hat on sunny days?**

- Yes
- No

**Do you use tanning beds (solarium)?**

- Yes
- No

**Do you regularly examine your skin and monitor skin lesions?**

- Yes
- No

**Do you sunbathe during holidays?**

- Yes
- No

**Do you go outside between 10:00 and 16:00 without sun protection?**

- Yes
- No

### **Attitudes Toward Sun Protection**

**I believe sun protection is important**

- True
- False

**I sometimes forget to use sunscreen**

- True
- False

**I do not have enough time during the day to use sunscreen**

- True
- False

**Sun protection is too expensive**

- True
- False

**I use sunscreen mainly to prevent aging rather than to prevent skin cancer**

- True
- False

### **Knowledge About Skin Cancer**

**Do you think you have sufficient knowledge about skin cancer?**

- Yes
- No

**Do you have knowledge about the main risk factors for skin cancer?**

- Yes
- No

**Do you have any knowledge about skin self-examination?**

- Yes
- No

**Can chronic skin irritation lead to skin cancer?**

- Yes
- No

**Do you know the most common anatomical locations of melanoma?**

- Yes
- No

**Do you know diagnostic methods for melanoma other than visual assessment?**

- Yes
- No

**Do you know the clinical features of skin cancers?**

- Yes
- No
